# PyMICE: APython library for analysis of IntelliCage data

**DOI:** 10.3758/s13428-017-0907-5

**Published:** 2017-06-22

**Authors:** Jakub M. Dzik, Alicja Puścian, Zofia Mijakowska, Kasia Radwanska, Szymon Łęski

**Affiliations:** 1 Department of Neurophysiology Laboratory of Neuroinformatics, Nencki Institute of Experimental Biology of Polish Academy of Sciences, Poland 3 Pasteur Str., Warsaw, 02-093 Poland; 2 Department of Neurophysiology Laboratory of Emotions Neurobiology, Nencki Institute of Experimental Biology of Polish Academy of Sciences, Poland 3 Pasteur Str., Warsaw, 02-093 Poland; 3 Department of Molecular and Cellular Neurobiology Laboratory of Molecular Basis of Behavior, Nencki Institute of Experimental Biology of Polish Academy of Sciences, Poland 3 Pasteur Str., Warsaw, 02-093 Poland

**Keywords:** Python, Library, Mice, Behavior, Analysis, IntelliCage

## Abstract

IntelliCage is an automated system for recording the behavior of a group of mice housed together. It produces rich, detailed behavioral data calling for new methods and software for their analysis. Here we present PyMICE, a free and open-source library for analysis of IntelliCage data in the Python programming language. We describe the design and demonstrate the use of the library through a series of examples. PyMICE provides easy and intuitive access to IntelliCage data, and thus facilitates the possibility of using numerous other Python scientific libraries to form a complete data analysis workflow.

## Introduction

In recent years, a number of automated environments for behavioral testing have been developed, based on RFID (Dell’omo, Shore, & Lipp, 1998; Galsworthy et al., 2005; Daan et al., 2011; Puścian et al., 2016) or video tracking of animals (de Chaumont et al., 2012; Weissbrod et al., 2013; Shemesh et al., 2013; Pérez-Escudero, Vicente-Page, Hinz, Arganda, & de Polavieja, 2014). Such automated systems have many advantages compared to the traditional behavioral tests, such as the reduction of the animal stress caused by isolation and handling (Heinrichs and Koob 2006; Sorge et al. 2014), the possibility of studying social interactions in group-housed animals (Kiryk et al. 2011), the possibility of long-term studies, and easier standardization of protocols in turn leading to better reproducibility of results between laboratories (Crabbe, Wahlsten, & Dudek, 1999; Chesler, Wilson, Lariviere, Rodriguez-Zas, & Mogil, 2002; Krackow et al., 2010; Codita et al., 2012; Morrison, 2014; Vannoni et al., 2014).

**Fig. 1:**
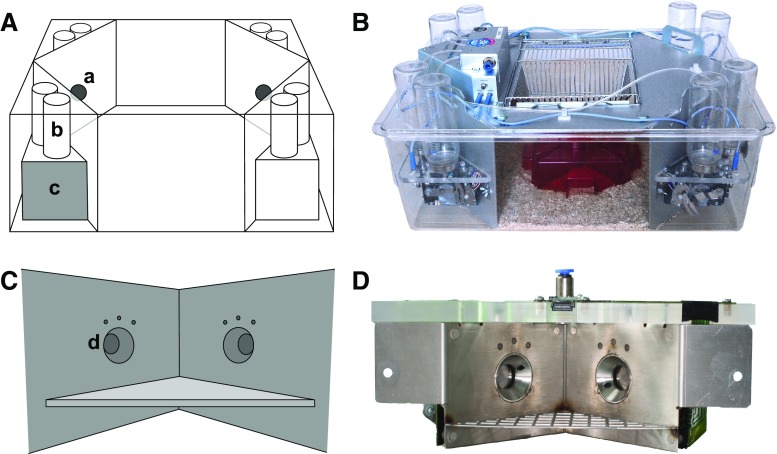
IntelliCage system. The system is composed of one or more cages (**A**, **B**). Through openings (**a**) mice can access bottles (**b**) in a learning chamber (**c**; **C**, **D**). Access to the bottles is controlled by programmable door in smaller openings in the sides of the chamber (**d**). *Credits:* **A**, **C** – Maria Nowicka, JD; **B** – Anna Mirgos, **D** – SŁ

The system we are particularly interested in is the IntelliCage system (NewBehavior A G 2011; Kiryk et al. 2011; Radwańska and Kaczmarek 2012; Puścian et al. 2014; Mijakowska et al. 2017) (see Fig. 1), which is increasingly popular in behavioral research on rodents (TSE Systems International Group 2016).

The system outputs a large amount of data describing the behavior of the mice in the conditioning corners of the cage. A typical experiment yields 10,000–100,000 visits recorded during several weeks or months. Such large data call for development of data analysis methods and software. One way to address the need of data analysis is to develop a dedicated application, preferably with a graphical user interface (GUI), which would allow researchers to inspect the data and extract relevant quantities interactively. In fact, such an application, called Analyzer, is provided with the IntelliCage system. While an interactive GUI-based program for data analysis may be useful, it does have certain limitations. In the context of scientific research, perhaps the most severe limitation is poor reproducibility of the analysis, unless strict measures are taken to record every single action of the user. Moreover, there is usually no automated way to perform exactly the same analysis on a different dataset, and repeating the analysis manually is very time-consuming and highly error-prone. Another inconvenience of ready-made programs is that they are typically limited to a predefined set of analysis methods, and not easily extendable.

Custom data analysis programs (e.g., scripts) fall at the opposite end of the spectrum than GUI programs. First, the most obvious advantage of such programs is the essentially unlimited possibility of implementing specialized analysis methods and applying them much better (in terms of calculation speed, precision and robustness) than a human.

Second, a noninteractive program (written in any programming language) running in batch mode is, by definition (Hoare 1969; Turing 1937; Floyd 1967; McCarthy 1963), an exhaustive specification of the analysis. In contrast, a plain language description usually included in a Methods section of a journal article may be ambiguous or not up-to-date. The voices calling for providing the data analysis workflows together with journal publications date at least two decades back (Buckheit and Donoho 1995): ‘an article about computational science in a scientific publication is not the scholarship itself, it is merely advertising of the scholarship. The actual scholarship is the complete software development environment and the complete set of instructions which generated the figures’.

Finally, one of the advantages of using an automated behavioral setup is the possibility of high-throughput screening by running the same protocol for a number of mice cohorts (for example treatment and control groups and/or different strains of mice (Puścian et al. 2014)). Manual processing of each dataset separately is both tedious and prone to errors. Batch-processing using a data analysis script is an obvious remedy, as it allows for repetition of exactly the same steps of analysis.

The only drawback is that the entry threshold for data analysis using a programming language is much higher, as significant effort is required to learn the programming language and necessary technical details (e.g., the data format). Our goal here is to lower this threshold by providing an easy and intuitive way to access the IntelliCage data in the Python programming language (van Rossum 1995).

This paper is organized as follows: first, we briefly describe the IntelliCage system. Next, we introduce PyMICE through a series of examples and provide pointers to further documentation. We conclude with a short section on technical details and a discussion.

## IntelliCage system

IntelliCage (Fig. 1) is an automated, computer-controlled RFID system for (possibly long-term) monitoring of groups of mice (Galsworthy et al. 2005; Krackow et al. 2010; Puścian et al. 2014). The mice are housed in one or more polycarbonate cages (of size 55 × 37.5 × 20.5 cm; Fig. 1A, B). A cage can house a group of up to 16 mice. Each mouse is tagged with an RFID transponder.

The key components of the system are learning chambers (Fig. 1c, C, D) located in the corners of cages (for brevity, we will refer to the chambers simply as ‘corners’). Each corner can be accessed through a circular opening (30 mm in diameter; Fig. 1a), with an embedded RFID antenna. By design, only one mouse at a time can enter a corner. Each corner contains two smaller (13 mm in diameter; Fig. 1d) openings, through which a mouse can access different drinking bottles (Fig. 1b). The access to the drinking bottles is controlled using programmable doors in the smaller openings.

A broad range of different experiment protocols can be implemented in the IntelliCage (Knapska et al. 2006; Kiryk et al. 2011; Endo et al. 2011; Radwańska and Kaczmarek 2012; Knapska et al. 2013; Smutek et al. 2014; Puścian et al. 2014; Vannoni et al. 2014). The system can be programmed to open the doors on specific conditions, for instance, if a specific mouse enters the corner or if a specific nosepoke pattern is performed. Also, an air puff in the back may be delivered to the mouse as a punishment.

The IntelliCage system records the visits of each mouse to a particular corner and also tracks the nosepokes—which lead to accessing the drinking bottles. When the mouse drinks from a bottle, the number of licks taken is also recorded. The events (visits and nosepokes) registered by the system are stored on a computer as a series of records. Each visit record contains, for example, the RFID transponder number of a given mouse, the cage and corner, and the time bounds of the visit. Further, nosepokes during the visit are also stored, along with time boundaries and the number of licks recorded, etc.

The system periodically logs the environmental conditions (ambient illumination and temperature) in every cage connected. It also logs other relevant events, such as: starts or ends of recording, errors, warnings, and hardware events (e.g., concerning state of the doors).

## PyMICE library

The IntelliCage system enables researchers to use sophisticated experimental protocols and thus explore behavioral phenomena inaccessible in classic, non-automated behavioral tests. However, in many cases, the analysis of data from such experiments poses a serious challenge. First, in some instances, manual analysis performed in the manufacturer’s software (Analyzer) would simply take too much time. For instance, analyzing data from experiments in which one is interested in a particular time frame with respect to a stimulus presented in the corner would be extremely time-consuming. If, for example, the availability of a reward is signaled by LEDs in a corner, and the diodes are only lit up in a specific time frame, then one would be naturally interested in, say, number of nosepokes performed before, during, and after the visual stimulus. Such information cannot be extracted from Analyzer in an automated way, and therefore the researcher would have to inspect each of the hundreds or thousands of visits manually. Another case in which it is hard to obtain the relevant data directly from Analyzer are protocols assessing impulsiveness of individual subjects by employing progressive ratio of behaviors (e.g. nosepokes) needed to obtain a reward. On the other hand, such protocols are useful for modeling symptoms of e.g. addiction (Radwańska and Kaczmarek 2012; Mijakowska et al. 2017).

A solution to this problem is to write custom software for automated data analysis. PyMICE is a free and open-source library that makes it easier to access and analyze IntelliCage data in the Python programming language.

Moreover, PyMICE is more suitable than the Analyzer software when it comes to the analysis of more sophisticated experimental designs (Endo et al. 2011; Knapska et al. 2013). Namely, it allows for the comprehensive analysis of variables than may not be computed in Analyzer. Publication of Knapska et al. from 2013 is an example of how highly specified behavioral assessment—in this case choosing between nosepoking to reward vs. to a neutral stimulus, performed right after entering a corner—might be implemented to identify neuronal circuits underlying specific cognitive deficits. The analysis in that paper was done manually, which required substantial effort. PyMICE facilitates drawing such conclusions by enabling experimenters with an easy access to highly specific parameters describing subjects’ behavioral performance.

For those reasons, we argue that PyMICE is a convenient solution to otherwise time-consuming data analysis and—more importantly—a valuable tool for in-depth analysis of previously inaccessible elements of murine behavior.

**Fig. 2:**
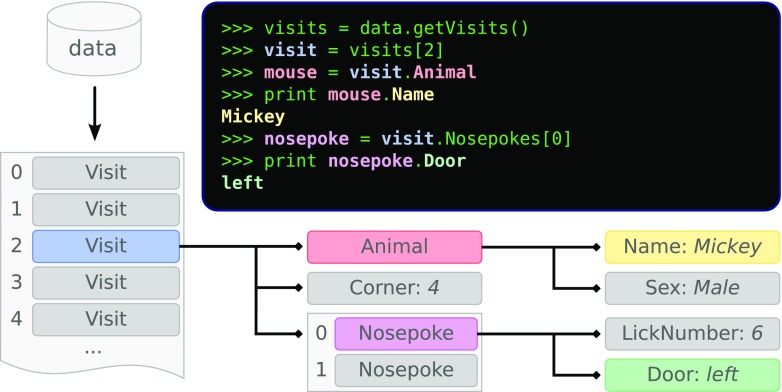
Visualization of PyMICE data structures. To investigate visit events recorded by the IntelliCage system, a list of Visit structures is obtained from the data object by the first command (*top right panel*). To focus on a third visit, the next command selects its item of index 2 (*pale blue*). To check the name of the mouse performing the visit, the third command accesses the .Animal attribute of the visit (*pale red*). The attribute is an Animal structure and the next command prints its .Name attribute (*yellow*). To further investigate which door was nosepoked during the visit, the .Nosepokes attribute (a tuple) must be accessed. The fifth command selects the first item (i.e., index 0) of the tuple, which is a Nosepoke structure (*pink*). The last command prints its .Door property (*pale green*)

One of the advantages of using the Python programming language is that a well-written Python program is readable to users. In fact, readability is stressed as one of the core Python principles (Peters 2004), which we have strived to follow in the design of PyMICE. Our library provides IntelliCage data as a collection of intuitively designed data structures (Fig. 2; Table 1), mirroring records written by the IntelliCage control software: most of the record fields are represented by attributes of the same or corresponding name. Also, auxiliary properties are provided, such as the .Door property of a Nosepoke object (see Fig. 2) translating integer value of the .Side attribute to ’left’ and ’right’ text strings. Manipulating such structures is straightforward and natural, therefore shifting the programmer’s focus from technical details of the file format to the data analysis itself. The data structures are read-only objects,^1^ which supports the functional programming paradigm.

**Table 1:** Data structures

IntelliCage data entity	Data structure (Python class)	Examples of attributes
Visit event	Visit	.Start, .Corner, .Animal, .Nosepokes
Nosepoke event	Nosepoke	.Start, .Side, .Visit
Sample of environmental condition	EnvironmentalConditions	.DateTime, .Illumination, .Temperature
Log entry	LogEntry	.DateTime, .Type, .Notes
Hardware event	HardwareEvent	.DateTime, .Type, .State
Animal	Animal	.Name, .Sex, .Tag
Group of animals	Group	.Name, .Animals

The data structures represent particular records written by the IntelliCage control software. Most fields are represented by attributes of the same or corresponding name

PyMICE operates on *ZIP* archives saved by the IntelliCage software controlling the experiment. Data from several recording sessions may be easily merged and analyzed together. All visits and nosepokes present in the raw data are loaded and presented to the user without any implicit filtering. Note that in some cases this leads to different results than those obtained with the Analyzer software bundled with the IntelliCage. One specific case we are aware of is that Analyzer (v. 2.11.0.0) omits some of the nosepokes present in the raw data, leading to potentially significant underestimation of the measured quantities (the worst case in the data we analyzed was 480 licks of a single mouse missing within a 6-h-long period of a liquid consumption study— 31*%* of the total recorded number).

The PyMICE library also facilitates automatic validation of the loaded data. A collection of auxiliary classes is provided for that purpose. Currently, possible RFID and lickometer failures may be detected automatically. Such events are reported in the IntelliCage logs, respectively, as *Presence Errors* and *Lickometer Warnings*. The set of detectable abnormal situations may be easily extended.

## Examples

In this section, we introduce PyMICE through a series of examples illustrating various aspects of the library.

In Example 1 we show how to find numbers of visits of a specific mouse in which the first nosepoke was performed to either the left side or the right side of the corner. This can be achieved in PyMICE in just six lines of code.

Example 2 is an extension of Example 1 to analysis of actual experimental data, obtained with a protocol described in Knapska et al. (2013). In this example, we also present a convention for defining the timeline of the experiment.

In Example 3 we reproduce a plot from an earlier paper (Puścian et al. 2014). The plot shows how two cohorts of mice learn the location of the reward over time (place preference learning). This kind of analysis can be performed using Analyzer, the GUI application provided with IntelliCage; however, using PyMICE we can quickly repeat the analysis for new cohorts with minimal effort.

Example 4 illustrates how the Python programming language can be used to extend the repertoire of data analysis methods. In this example we show how to extract the information about intervals between visits of different mice to the same corner. This kind of information would be very hard (or even impossible) to obtain using Analyzer.

To improve readability of Examples 2–4, we have omitted some generic code and focused on PyMICE-specific snippets. Full code of the examples is provided as online supplementary material at https://github.com/Neuroinflab/PyMICE_SM/tree/examples.

Before the examples can be run, the example data have to be saved to the current working directory. This can be done from within PyMICE:

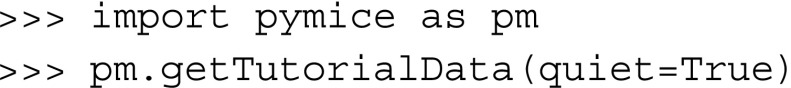



In addition to the examples presented here, we have prepared several tutorials available online at the PyMICE website (Dzik and Łęski 2017b). The tutorials are in the Jupyter Notebook (Project Jupyter 2015) format and may be downloaded for interactive use. The examples and the tutorials are provided as a hands-on introduction to PyMICE and serve as a starting point for further exploration. Additionally, online documentation is provided (Dzik and Łęski 2017a). PyMICE objects and their methods are also documented with docstrings available with Python built in help() function.

### Example 1: minimal example—extracting the side of the first nosepokes in six lines of code

This example shows how to obtain the number of visits in which a mouse performed the first nosepoke to the left side or the right side of the corner.

In the first line, the library is imported. We use pm as an abbreviated name of the library. In the second line, the example data are loaded from a single IntelliCage file. In the third line, a list of all visits of a mouse named ‘Jerry’ is obtained.^2^ Note that a list of names is passed as an argument.

Next, the first nosepoke is selected from every visit v (if any nosepoke was made during that visit). Note that the condition if v.Nosepokes is met if the list v.Nosepokes is not empty.

The side the mouse poked is obtained as nosepoke. Door in the fifth line of the code. This returns either ’left’ or ’right’, thus disregarding the information about the corner in which the nosepoke was performed.

Finally, in the last line, the number of visits with the first nosepoke to the left and the right side is calculated and displayed.

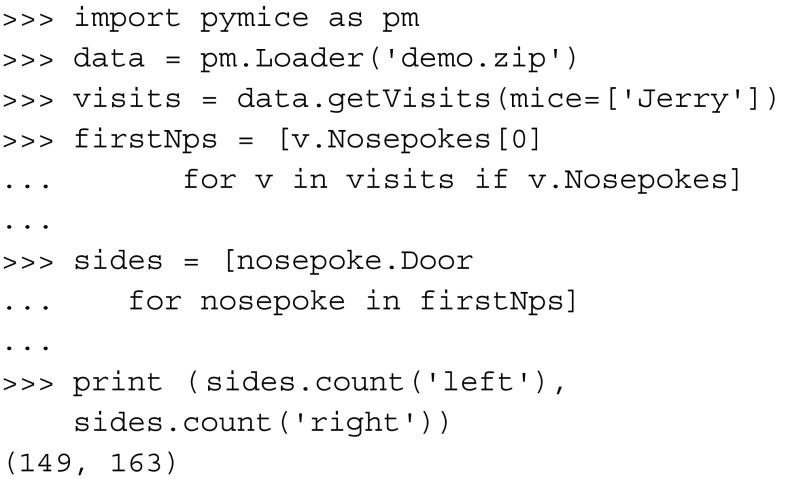



### Example 2: full analysis example—side discrimination task

The previous example is a simplified version of an analysis performed to assess place memory during the discrimination training, as described in Knapska et al. (2013). In this example, we present the analysis in more detail, including the estimation of how the mice performance in the discrimination task changed over time.

The experimental setup was the following: during the first several days of the experiment, the mice were adapted to the cage. In this phase, water was freely available in all corners, at both sides of each corner, and the doors to the bottles were open. Next, in nosepoke adaptation (*NPA*) phase of the experiment, mice had to perform nosepokes to access the water bottles. The next phase was place preference learning (*PP*). In this phase, every mouse could access bottles in one corner only. The mice were assigned to different corners as evenly as possible to prevent crowding and learning interference. The final experimental phase was the discrimination task (*DISC*). In this phase, the mice were presented with two bottles in the same corner to which they were assigned during the *PP* phase. However, in contrast to the *PP* phase, one bottle contained tap water, and the other contained 10% sucrose solution (highly motivating reward). As previously, during each visit the access was granted to both bottles. The percentage of visits in which the first nosepoke was performed to the bottle containing reward was calculated as a measure of memory.

We start by loading the data. Note that some technical details not crucial for understanding the example (like importing the libraries) are hidden. The full code is available at https://github.com/Neuroinflab/PyMICE_SM/blob/examples/example2.py.

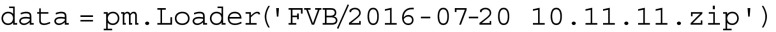



Next, we need to know the start- and end-points of the experimental phases we are interested in. We defined these phases in an experiment timeline file. The format of this file is a derivative of the *INI* format. The necessary information required about a phase are: its name (*PP dark* in the following example), boundaries (start and end properties) and timezone of the boundaries (tzinfo property):

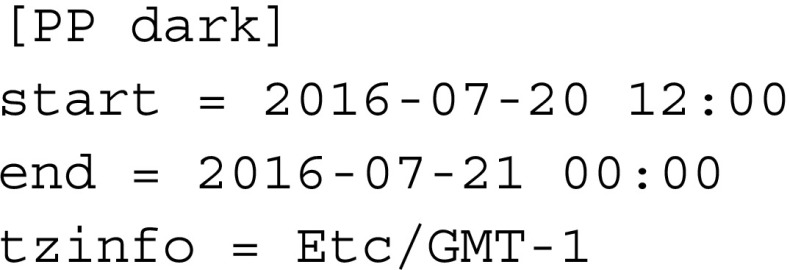



We load the experiment timeline file into a Timeline object, and define a list of phases of interest.

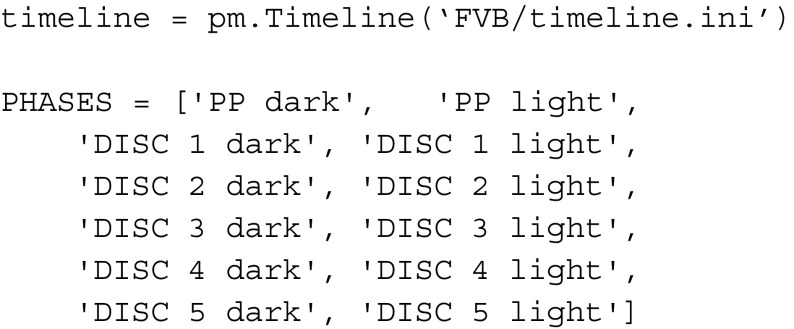



To analyze the data, we define a function get PerformanceMatrix(), which returns a matrix of performance (defined as the fraction of first nosepokes in the accessible corner, which are performed to the rewarded side) of all mice in the system. Each row in this matrix contains the performance data of one mouse, and each column corresponds to one phase of the experiment for all mice.

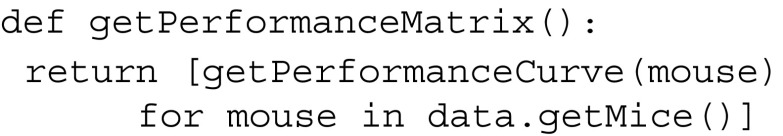



For every mouse, the getPerformanceCurve() function is called to create a row corresponding to the performance of that mouse across subsequent phases.

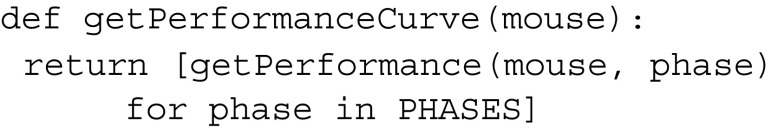



We use the getPerformance() function to create a list of the mouse’s performance measures (fraction of first nosepokes in the accessible corner, which are performed to the rewarded side) in subsequent phases.

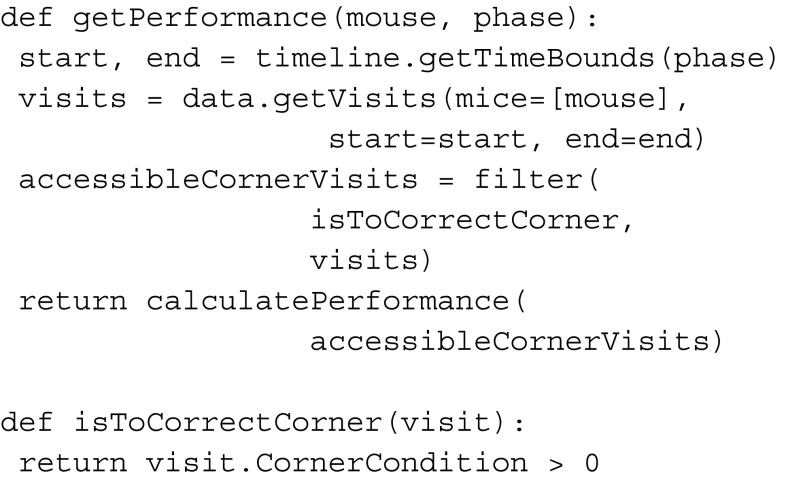



In the getPerformance() function, we obtain all visits performed by the mouse during the phase. Visits to the accessible corner are passed to the calculate Performance() function for further analysis. It is easy to filter the visits—in the IntelliCage experiment design the accessible corner was marked as ‘correct’. Thus, visits to the corner have positive value of the .CornerCondition attribute of the Visit object.

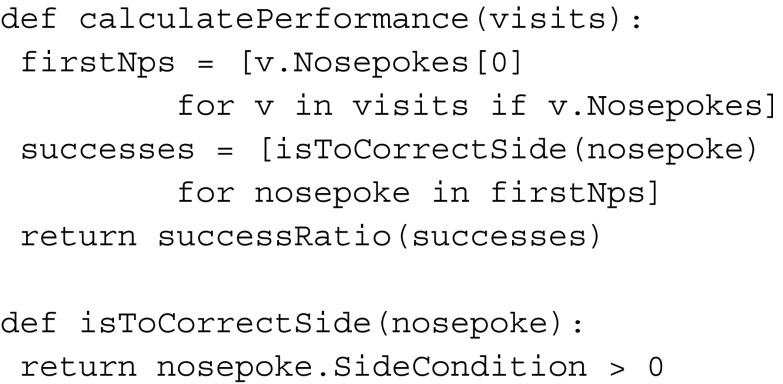



To calculate the performance, we check whether the first nosepoke of every visit was to the rewarded side. The rewarded side was marked as ‘correct’ in the experiment design. Thus, nosepokes to the correct side have positive value of the .SideCondition attribute of the respective Nosepoke object. The side which was rewarded in *DISC* phases was marked as ‘correct’ already during the *PP* phases (when both bottles in a corner contained the same liquid), so that the relevant fraction can be easily extracted also from the *PP* phases.

Based on the counts of first nosepokes performed to each of the sides, we calculate the success ratio (code omitted here).

**Fig. 3:**
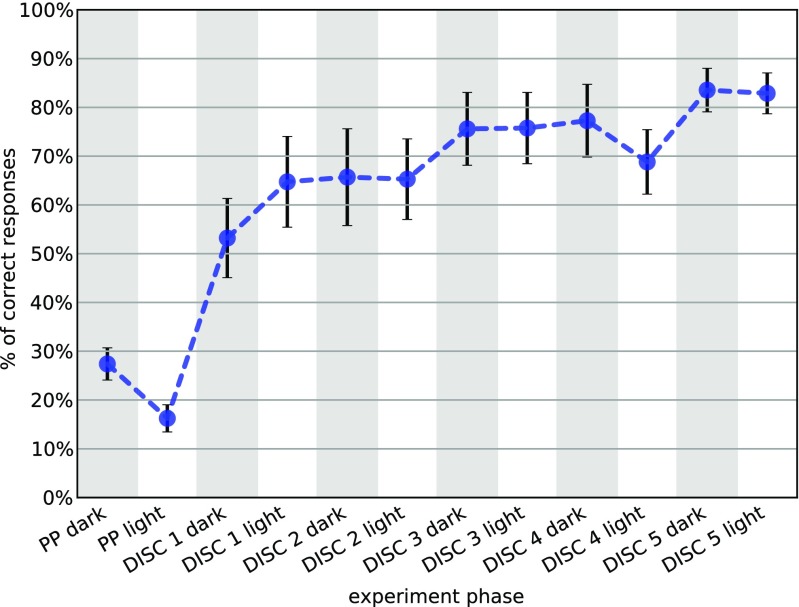
Reward-motivated discrimination learning in FVB mice (n = 11). The chart depicts efficient learning of reward position, as measured by the percentage of first nosepokes made to the bottle containing reward right after entering the corner. *Error bars* are standard error of the mean

With all the functions defined, we obtain the performance matrix and plot it averaged across mice in Fig. 3 (plotting code omitted here).

### Example 3: reproducibility—batch analysis of data

In this example, we analyze results of a place preference experiment described in detail in Puścian et al. (2014). Similarly as in Example 2, the experiment comprised several adaptation and learning phases, in which the mice could access either all or just selected drinking bottles. In the nosepoke adaptation phase (*NPA*), all the mice had access to tap water in all corners. In order to obtain water, the mice were required to open the door by performing a nosepoke. Next, in the place preference learning phase (*Place Pref*), the access to the drinking bottles was (as in the previous example) restricted to just one corner for each mouse. Tap water was replaced with 10% sucrose solution to increase the motivation of the mice to seek access to the drinking bottles. We are interested in how the percentage of visits to the rewarded corner changed over time.

**Fig. 4:**
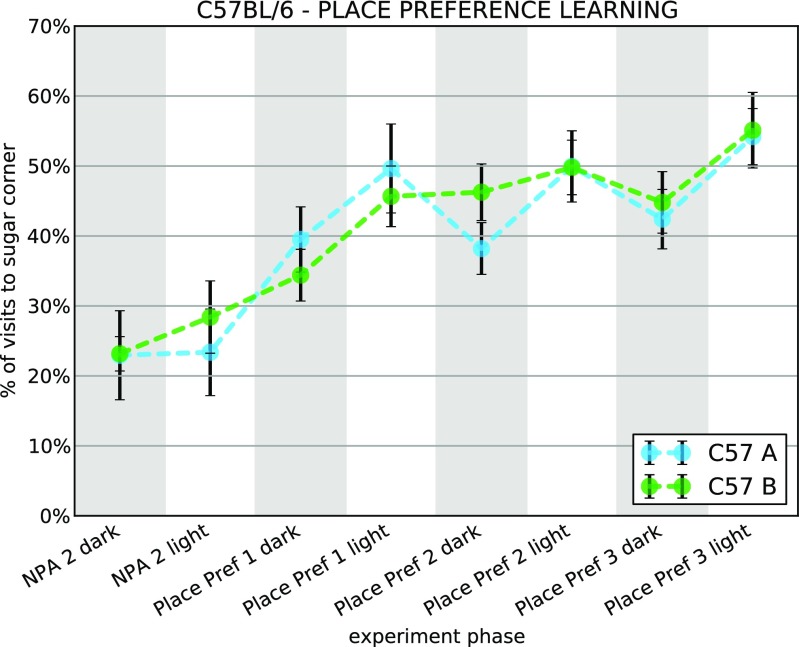
Place preference learning of two cohorts of C57BL6 mice in an IntelliCage experiment. The plot presents the cohort-averaged percentage of visits to a rewarded corner during consecutive phases, each lasting 12 h. During the place preference phases (Place Pref), each mouse is provided access to sweetened water in one selected corner of the IntelliCage. The fraction of visits to that corner is close to the chance level (25%) during the NPA (nosepoke adaptation) phases when mice are provided access to plain water in all corners, and increases over time after the reward (sweetened water) is introduced. *Error bars* are standard error of the mean

The data used here are a subset of data presented in Puścian et al. (2014), and in the final figure obtained in this example (Fig. 4) we show two learning curves from Fig. 3A in Puścian et al. (2014) (cohorts A and B). Each curve represents an average performance (defined as a fraction of visits to the rewarded corner) of a cohort of mice in eight subsequent, 12-h-long phases of the experiment.

As the code here is quite similar to the code of the previous example, we will focus on the major differences between the examples. We start by loading the data, timeline and PHASES objects from the relevant dataset (different than in Example 2; code omitted here, the full code is available at https://github.com/Neuroinflab/PyMICE_SM/blob/examples/example3.py).

As in the previous example, we define a function returning a performance matrix (defined here as the fraction of visits to the rewarded corner): performanceMatrix().

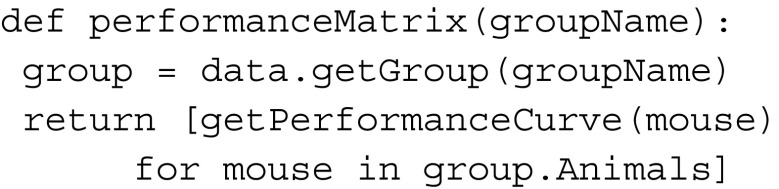



Unlike in the previous example, the matrix here is limited to only one group of mice. The group is defined in the IntelliCage experiment design. Information about its members is contained in a Group object, which we request in the first line of the function.

The getPerformance() and calculatePerformance() functions are simpler than in the previous example, as neither filtering of visits nor extracting of nosepokes is necessary.

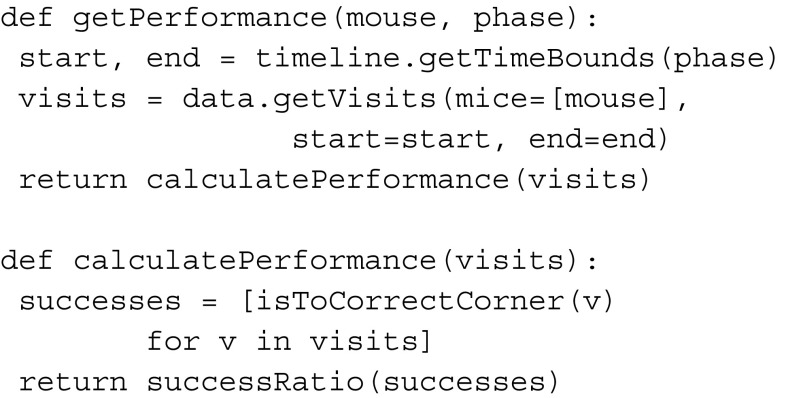



To calculate the performance of a mouse during a phase, we check—for each visit—whether the visit was to the rewarded corner. In the IntelliCage experiment design, the rewarded corner was marked as ‘correct’. Visits to the ‘correct’ corner have positive value of the .CornerCondition attribute of the Visit object.

With all the functions defined, we obtain a performance matrix and plot it averaged across mice (Fig. 4) for each cohort (groups C57A and C57B) separately. Note that analyzing several cohorts reduces to a loop over the cohorts included in the analysis.

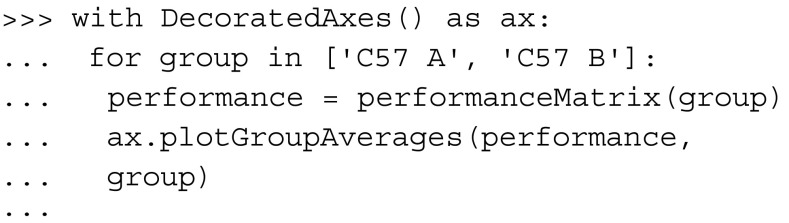



### Example 4: implementing new behavioral measures—intervals between visits

In this example, we illustrate the possibility of programming new data analysis methods in Python. We are going to investigate durations of intervals between subsequent visits of mice to a corner. The assumption here is that the distribution of such intervals is a measure of interactions between the mice. In this example we will just calculate the measure without discussing it much, but we believe that such measure, or a variant of it, would be useful in studying social behaviors or social structure of the group. In particular, one could study which mice follow which, and therefore look into potential modulation of learning or cognitive abilities by such behaviors as following or imitation.

We want to plot histograms of interval durations for each corner separately, and we restrict the analysis to just one phase: *Place Pref 3 dark*. Such analysis would be very hard or impossible to perform using Analyzer, and requires the use of some kind of programming language, which is the main reason we include it in the paper. Below we show that the analysis in Python using PyMICE is quite straightforward.

We begin by selecting all visits performed during the phase. The order parameter of the .getVisits() method makes the returned sequence ordered with respect to the .Start attribute.

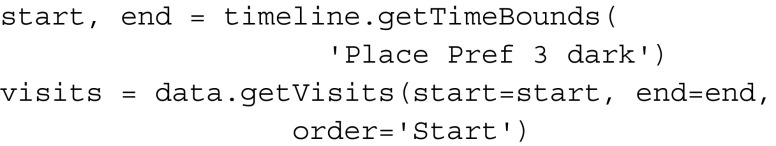



This list contains visits performed to all corners of the cage. Next we need to extract subsequences of visits performed to the same corner in the same cage.

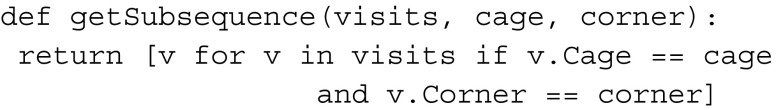



Since the order of the subsequence is preserved, it is then easy to determine intervisit intervals.

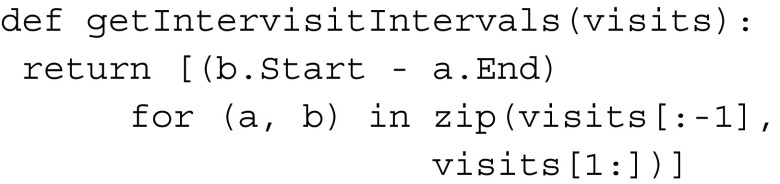



**Fig. 5:**
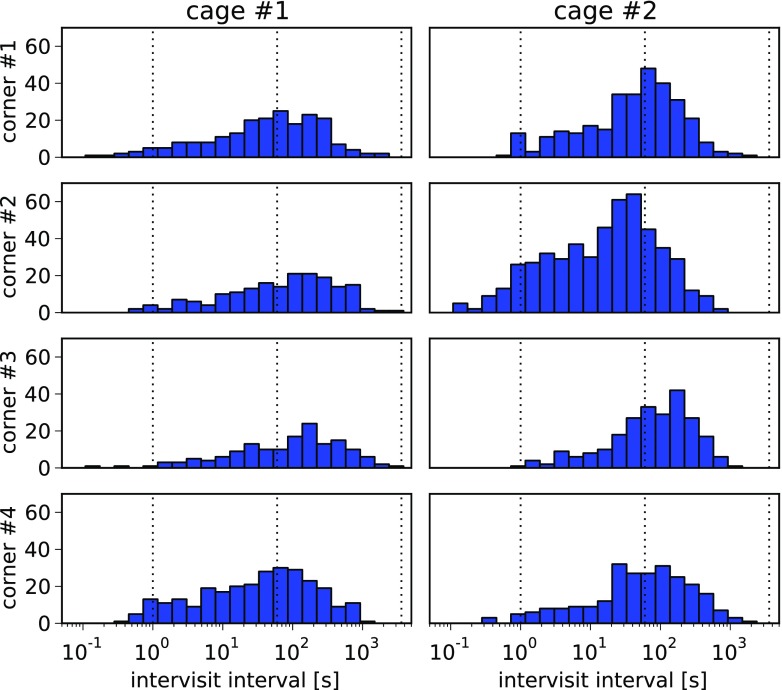
Frequencies of intervisit intervals in the analyzed phase. Histograms are plotted for each corner of each cage separately. The bins are equally spaced in a logarithmic scale. The *dotted vertical lines* represents intervals of one second, minute and hour (from *left* to *right*, respectively)

The histograms are shown in Fig. 5. As in the previous examples, the details of plot generation are hidden. The full code is available at https://github.com/Neuroinflab/PyMICE_SM/blob/examples/example4.py.

## Technical details

We recommend to use the PyMICE library with the Anaconda Python distribution (Continuum Analytics 2015).

The library requires *NumPy* (van der Walt, Colbert, & Varoquaux, 2011; Oliphant, 2007), *matplotlib* (Hunter 2007), *dateutil* (Niemeyer, Pieviläinen, & de Leeuw, 2016) and *pytz* (Bishop 2016) Python packages to be installed in the system.

The library itself is available as a package from the Python Package Index (PyPI) (Jones 2002; Python Software Foundation 2016) for Python version 3.3, 3.4, 3.5 and 3.6, as well as 2.7. It can be installed with either *pip* (Python Packaging Authority 2016): 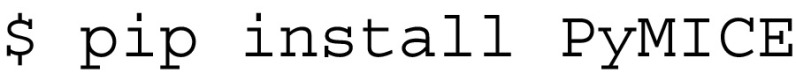


or *easy_install*(Python Packaging Authority 2017): 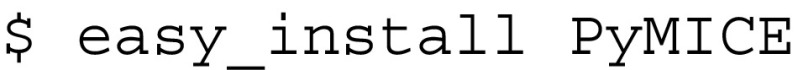


A bleeding edge version of the library might be also downloaded from https://github.com/Neuroinflab/PyMICEGitHub repository.

## Terms of use

PyMICE library is open-source and is available for free under GPL3 license (Free Software Foundation 2007); we ask that this article is cited and resource identifier (Ozyurt et al. 2016) for the library (*RRID:nlx_158570*) is provided in any published research making use of PyMICE.

## Discussion

In this paper we have introduced PyMICE, a software library which allows to access and analyze data from IntelliCage experiments. The library has been developed to facilitate automated, reproducible, and customizable analysis of large data generated by the IntelliCage system. Analyzer, the software bundled with the IntelliCage, does not meet these requirements, as it was designed with a different purpose in mind (NewBehavior A G 2011): ‘The “Analyzer” is intended to give an overview of the results [...] The function of “Analyzer” is to provide the user with data merging, extraction, and filtering tools in order to generate data sets appropriate for in-depth graphical and statistical analyses.’

One of the features of the IntelliCage system is that very different experiments are possible, depending on the subject of the research. Some protocols focus on assessment of subjects’ ability of reward location (Knapska et al. 2013) and behavioral sequence (Endo et al. 2011) learning. Other protocols are dedicated to measure addiction-related behavior like subject impulsiveness (Radwańska and Kaczmarek 2012; Mijakowska et al. 2017). Quite often a new experiment requires a completely new approach to data analysis. Rather than trying to predict the specific needs of the prospective users, we decided to provide simple, intuitive and user-friendly interface for accessing the data. Such interface allows a scientific programmer to tailor dedicated software focusing on the essence of the analysis instead of the technical details. To our knowledge, PyMICE is the only freely available solution for analysis of IntelliCage data in a scripting language.

PyMICE is written in the Python programming language. Our choice of Python was directed by the same factors which made it a popular choice for scientific computing in general. Python is free, open-source, relatively easy to learn, and is supported by a number of scientific tools and libraries, such as: NumPy and SciPy (Oliphant 2007), IPython (Perez and Granger 2007), matplotlib, Pandas (McKinney 2010), etc. We believe that PyMICE will be a useful addition to that collection.

The number of IntelliCage-based publications is increasing in recent years (TSE Systems International Group 2016), but the system is still relatively little known. We believe that one of the factors handicapping the popularity of IntelliCage, or similar automated setups, is the lack of a proper software ecosystem. We hope that the availability of PyMICE will have a stimulating effect on the adoption of automated behavioral systems. While the current (at the time of the publication) version of the library only supports the IntelliCage, the library may be generalized to other behavioral systems. Data from any system capturing point events (such as visits to specific locations—as opposed to e.g. continuous trajectories of the animals) could be presented to the user in a similar way as the IntelliCage data. Specifically, representing each behavioral event as a Python object with relevant attributes would allow for intuitive manipulation of data and for easy extraction of the quantities which are analyzed. The PyMICE library is open source (Free Software Foundation 2007) and publicly available at GitHub, the largest open source software platform (Gousios, Vasilescu, Serebrenik, & Zaidman, 2014), therefore the extensions to other behavioral systems can be contributed by the community.

A crucial feature of PyMICE is the possibility of creating automated data analysis workflows. Such workflows are useful, for example, when the same protocol is applied to multiple groups of animals—this is a very common case, as most experiments will have at least one experimental and one control group. A workflow defined in a Python script may be used to perform exactly the same analysis on every available dataset, which both saves effort and greatly reduces possibility of mistakes as compared to analyzing each dataset manually.

We also believe that popularization of such workflows would lead to better research reproducibility. Current efforts for reproducible research are mostly focused on improving the experimental procedures, statistical analysis, and the publishing policy (Begley & Ellis, 2012; Begley, 2013; Halsey, Curran-Everett, Vowler, & Drummond, 2015). However, unclear or ambiguous description of data analysis is also given as a factor contributing to poor reproducibility of scientific research (Ince, Hatton, & Graham-Cumming, 2012). A (non-interactive) computer program is a precise, formal and unambiguous description of the analysis performed. We hope that PyMICE could become a common platform for implementing and sharing workflows for analysis of data from the IntelliCage (or similar) system, and make data analysis using scripts more accessible and more popular.

The paper itself is a proof of the concept of the ‘really reproducible’ research (Buckheit and Donoho 1995)—writing it we followed the literate programming paradigm (Knuth 1984). Every of the presented results of analysis was generated by a Python + PyMICE workflow embedded in the LATE X (Lamport 1986) source code of the document (see the *Statement of reproducibility* below for details).

## Statement of reproducibility (Peng 2011)

The source code of the paper is available at https://github.com/Neuroinflab/PyMICE_SM/. Paper compiled from the source code may differ from the journal version because of manual formatting. All presented results may be reproduced with the *Pweave* tool (Pastell, 2015) v. 0.24, (Pastell and Kowalski 2016) an interpreter of *Python* programming language v. 2.7.3, *PyMICE* v. 1.1.0, (Dzik, Łęski & Puścian, 2016) *NumPy* v. 1.6.1, *matplitlib* v. 1.1.1rc, *dateutil* v. 1.5 and *pytz* v. 2012c. (Wilson et al. 2014).

## Footnotes

1 The two exceptions are: the Animal semi-mutable class (the .Sex attribute might be updated if None; the .Notes attribute might be extended) and the Group mutable class.

2 The mice are referred to by names defined in the design of IntelliCage experiment, not by the RFID tag numbers.
